# Are the intrinsically disordered linkers involved in SSB binding to accessory proteins?

**DOI:** 10.1093/nar/gkz606

**Published:** 2019-07-22

**Authors:** Min Kyung Shinn, Alexander G Kozlov, Binh Nguyen, Wlodek M Bujalowski, Timothy M Lohman

**Affiliations:** 1 Department of Biochemistry and Biophysics, Washington University in St. Louis School of Medicine, St. Louis, MO 63110, USA; 2 Department of Physics, Washington University in St. Louis, St. Louis, MO 63130, USA; 3 Department of Biochemistry and Molecular Biology, The University of Texas Medical Branch, Galveston, TX 77555, USA

## Abstract

*Escherichia coli* single strand (ss) DNA binding (SSB) protein protects ssDNA intermediates and recruits at least 17 SSB interacting proteins (SIPs) during genome maintenance. The SSB C-termini contain a 9 residue acidic tip and a 56 residue intrinsically disordered linker (IDL). The acidic tip interacts with SIPs; however a recent proposal suggests that the IDL may also interact with SIPs. Here we examine the binding to four SIPs (RecO, PriC, PriA and χ subunit of DNA polymerase III) of three peptides containing the acidic tip and varying amounts of the IDL. Independent of IDL length, we find no differences in peptide binding to each individual SIP indicating that binding is due solely to the acidic tip. However, the tip shows specificity, with affinity decreasing in the order: RecO > PriA ∼ χ > PriC. Yet, RecO binding to the SSB tetramer and an SSB–ssDNA complex show significant thermodynamic differences compared to the peptides alone, suggesting that RecO interacts with another region of SSB, although not the IDL. SSB containing varying IDL deletions show different binding behavior, with the larger linker deletions inhibiting RecO binding, likely due to increased competition between the acidic tip interacting with DNA binding sites within SSB.

## INTRODUCTION

**Figure 1.**
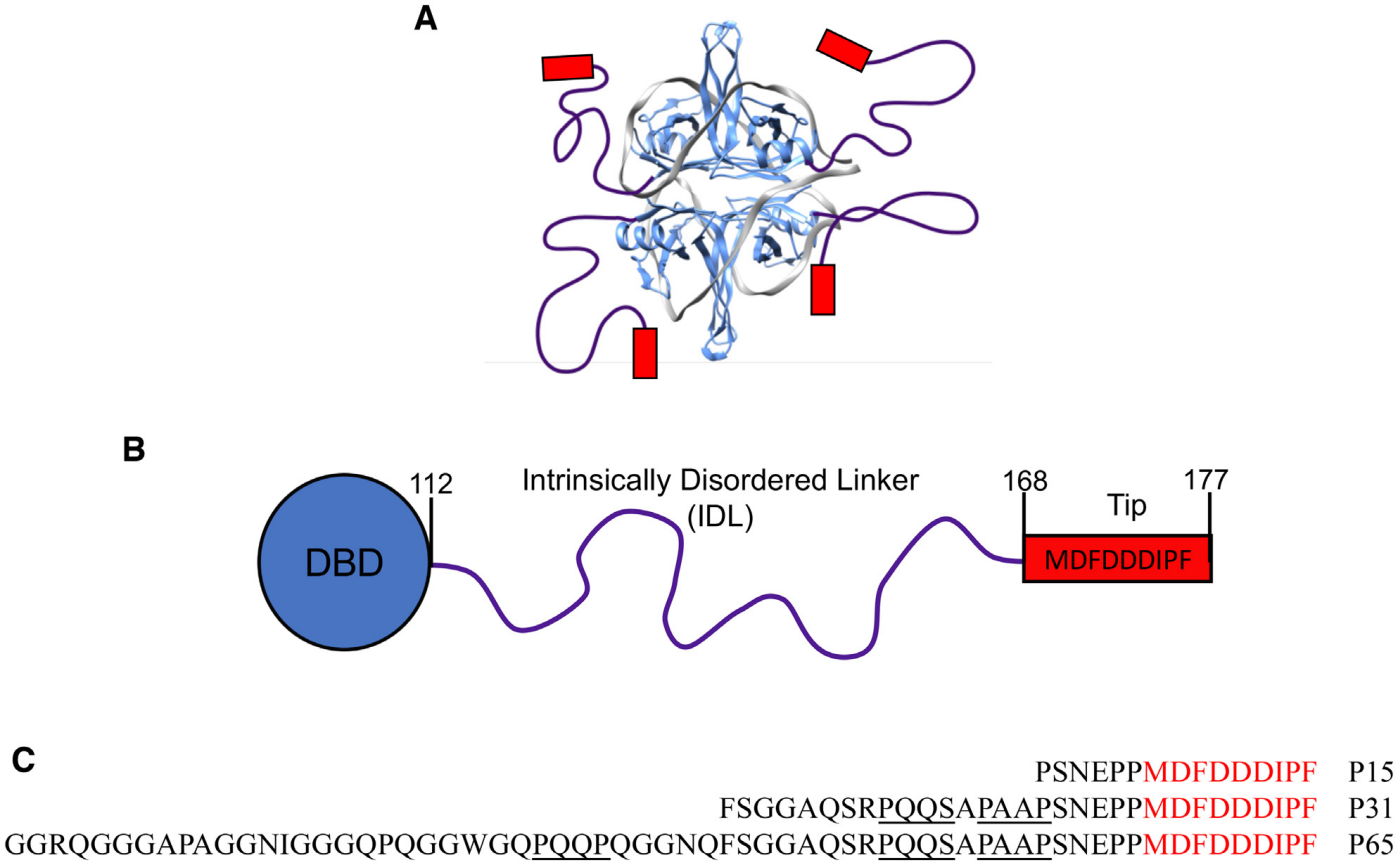
Structure of *Escherichia coli* SSB. (**A**) Model for the structure of an SSB tetramer complexed with (dT)_70_ in the (SSB)_65_ binding mode (1). The IDL (purple lines) and the nine amino acid acidic tips (red boxes), are shown schematically. (**B**) A cartoon representation of the domain structure of an SSB subunit showing the N-terminal DNA binding domain (DBD) (residues 1–112), the 56 amino acid IDL (residues 113–168) and the acidic tip (residues 169–177). (**C**) Sequences of the SSB-Ct peptides with the PXXP motifs underlined in black.

*Escherichia coli* single stranded (ss) DNA binding protein (SSB) forms a homo-tetramer (1) with each subunit possessing two domains, an N-terminal ssDNA binding domain (residues 1–112) and a C-terminal domain consisting of a 56 amino acid intrinsically disordered linker (IDL) (residues 113–168) and a 9 amino acid ‘acidic tip’ (residues 169–177) (Figure 1). *Escherichia coli* SSB plays two essential roles in genome maintenance: to bind and stabilize ssDNA intermediates (2–6), and to act as a hub to recruit more than 17 proteins involved in DNA recombination (7–19), replication (20–24), replication restart (25–28) and repair (29–37). SSB binds non-specifically to ssDNA with high affinity in multiple DNA binding modes depending on solution conditions. Two of the major binding modes are the (SSB)_35_ and (SSB)_65_ modes, where the subscripts denote the average number of ssDNA nucleotides occluded per SSB tetramer (38–40). The partially wrapped (SSB)_35_ mode in which an average of only two subunits interact with ∼35 nt of ssDNA is promoted at low monovalent salt concentrations (<10 mM [NaCl]) and high SSB to ssDNA ratios, and exhibits unlimited inter-tetramer cooperativity resulting in the formation of protein clusters (40–43). The fully wrapped (SSB)_65_ mode, in which all four subunits bind ssDNA, is promoted at higher monovalent salt concentrations (>200 mM NaCl) and displays only limited cooperativity with little protein clustering (38,39,41–48). An intermediate (SSB)_56_ mode can also form (39), but less is known of its properties.

*Escherichia coli* SSB interacts via its acidic tip with at least 17 other proteins referred to as SSB interacting proteins (SIPs) (5). The potential roles for the IDL have only recently begun to be uncovered. In contrast with the highly conserved DBD of bacterial SSB proteins, the IDL is not highly conserved and varies in length from ∼25 to 125 amino acids, with *E. coli* SSB having an IDL of 56 residues. The *E. coli* SSB IDL is essential for its highly cooperative binding to long ssDNA (49,50), that seems to involve non-nearest neighbor SSB interactions (51), although interactions involving residues within the DBD have also been identified that affect cooperativity (52,53). The amino acid content of the IDL is clearly important for cooperativity since replacing the IDL with the more highly charged IDL from *Plasmodium falciparum* SSB eliminates highly cooperative binding (49). SSB variants in which the length and/or number of C-terminal tails have been modified affect both cooperativity and the relative stability of the SSB-DNA binding modes, with deletion of the IDL (50) or removal of two or three tails favoring the (SSB)_35_ mode (54). At least two SSB tails are required for a functional SSB tetramer *in vivo* (54).

Since SSB interacts with so many SIPs, a major question involves whether and how SSB can differentiate and regulate its binding to the different SIPs and what is the basis for any specificity. Although there is a large amount of data indicating that the acidic tip of the SSB C-termini provides the major site of interaction with the SIPs, it has recently been suggested that the site of interaction of SSB with at least some SIPs is contained within the IDL and not the acidic tip (55,56). Specifically, it has been proposed that the three proline-rich PXXP motifs contained within the IDL interact with the OB-folds of some SIPs in a manner analogous to the motifs binding to SH3 domains. Hence the IDL could potentially contribute to both affinity and specificity. In this report we compared the energetics of binding of four *E. coli* SIPs: *E. coli* RecO, a recombination mediator involved in the RecF pathway of DNA repair (14,57,58), the chi (χ) subunit of DNA Pol III (59), *E. coli* PriA and PriC which function in replication restart (28,60), to a series of peptides containing the acidic tip and various lengths of the IDL. RecO binding to SSB variants containing linker deletions alone and in complex with ssDNA was also examined.

## MATERIALS AND METHODS

### Buffers and reagents

Buffers were prepared with reagent grade chemicals using distilled, deionized water (Milli-Q system; Millipore corp., Bedford, MA, USA). Spectrophotometric grade glycerol was from Alfa Aesar (Ward Hill, MA, USA). Buffer BTP is 20 mM Bis-Tris Propane (pH 8.0 at 25°C), 25% (v/v) glycerol, 1 mM TCEP. Buffer T is 10 mM Tris–HCl (pH 8.1 at 25°C), 200 mM NaCl and 0.1 mM ethylenediaminetetraacetic acid.

### Proteins, peptides and DNA

*Escherichia coli* RecO protein was overexpressed from plasmid pMCSG7 in *E. coli* strain BL21(DE3)pLysS (kindly provided by Dr Sergey Korolev) and purified using Ni-NTA affinity chromatography and a HiTrap Heparin HP affinity column (GE Healthcare, Chicago, IL, USA) after His-tag cleavage with TEV protease as described (19). The autoinactivation-resistant S219V mutant of TEV protease with an N-terminal His-tag and C-terminal polyarginine tag (His-TEV(S219V)-Arg) was overexpressed from *E. coli* strain BL21(DE3) transformed with PRK793 and pRIL (Stratagene, San Diego, CA, USA) and purified as described (61). *Escherichia coli* SSB and the deletion mutants were purified as described (62,63). The SSB-A construct has an additional six amino acids, TGASGT, extending the C-terminus of wtSSB. χ protein and PriA were expressed and purified as described (64). PriC was overexpressed from *E. coli* strain BL21(DE3) transformed with plasmid pET11a-EcoPriC (pAM001) (kindly provided by Dr. James Keck) and purified as described (65), but with Macro-Prep High S Resin instead of SPFF (Bio-Rad, Hercules, CA, USA) and HiPrep Sephacryl S-200 HR column (GE Healthcare Bio-Sciences, Pittsburgh, PA, USA) instead of S100. The concentrations of RecO, PriA, χ and PriC were determined using extinction coefficients of ϵ_280_ = 2.44 $ \times$ 10^4^ M^−1^ cm^−1^, ϵ_280_ = 1.06 $ \times$ 10^5^ M^−1^ cm^−1^, ϵ_280_ = 2.92 $ \times$ 10^4^ M^−1^ cm^−1^ and ϵ_280_ = 2.39 $ \times$ 10^4^ M^−1^ cm^−1^, respectively. The concentrations of wtSSB and SSB constructs in units of tetramers were determined using extinction coefficients of ϵ_280_ = 1.13 $ \times$ 10^5^ M^−1^ cm^−1^ for wtSSB, SSB-A, SSBΔ151-166 and ϵ_280_ = 8.98 $ \times$ 10^4^ M^−1^ cm^−1^ for SSBΔ130-166 and SSBΔ120-166. The sequences of the C-termini of the SSB deletion mutants are shown in Figure 5A.

SSB-Ct peptides, P15, P31 and P65, corresponding to the C-terminal 15, 31 and 65 amino acids of SSB, were purchased from WatsonBio (Houston, TX, USA). The sequences of the peptides are shown in Figure 1C. The concentrations of the peptides were determined using an extinction coefficient of ϵ_258_ = 390 M^−1^ cm^−1^ (66) for P15 and P31 based on the two Phe residues and ϵ_280_ = 5500 M^−1^ cm^−1^ for P65 based on the single Trp residue. The (dT)_70_ was synthesized and purified as described (40), and the concentration was determined in units of nucleotides using the extinction coefficient ϵ_260_ = 8.1 × 10^3^ M^−1^ cm^−1^ (67).

### Isothermal titration calorimetry (ITC)

Isothermal titration calorimetry (ITC) experiments were performed using a VP-ITC titration microcalorimeter (Malvern Panalytical, Malvern, UK) (68). All proteins and oligonucleotides were dialyzed extensively against the indicated buffer and cleared by centrifugation at 14 000 rpm for 15 min at 4°C. The concentrations were determined thereafter. For experiments with the SSB-Ct peptides and SIPs, 40–50 μM peptides were titrated into 1–2 μM SIPs in Buffer BTP at pH 8.0 and 50 mM NaCl. For experiments with the full-length SSB and its deletion mutants and SIPs, SSB tetramer (8–10 μM) was titrated into SIP (1 μM) in Buffer BTP at pH 7.0 and 200 mM NaCl. The heats of dilution were obtained by blank titrations in which the titrant species is titrated into the buffer, and corrections for heats of dilution were applied.

The raw data were analyzed to obtain titration curves by integrating each peak from the time of titrant addition until equilibration back to the baseline using ‘MicroCal Data Analysis’ software provided by the manufacturer. The binding parameters, stoichiometry (*N*), observed association equilibrium constant (${K_{obs}}$) and binding enthalpy change (${\rm{\Delta }}{H_{obs}}$), were obtained by fitting the titration curves to a model of ligand (X, SSB-Ct peptides or RecO) binding to *N* identical and independent sites on the macromolecule (M, SIPs or SSB) using Equation (1),

(1) $$\begin{equation*}Q_i^{{\rm tot}} = {V_0}{\rm{\ \Delta }}{H_{{\rm obs}}}{M_{{\rm tot}}}\frac{{N{K_{{\rm obs}}}X}}{{1 + {K_{{\rm obs}}}X}}\end{equation*}$$

where $Q_i^{{\rm tot}}$ is the total heat after the *i*th injection and ${V_0}$ is the volume of the calorimetric cell. The concentration of the free ligand (X) was obtained by solving Equation (2).

(2) $$\begin{equation*}{X_{{\rm tot}}} = \ X + \ {X_{{\rm bound}}} = \ X + \frac{{N{K_{{\rm obs}}}X}}{{1 + {K_{{\rm obs}}}X}}{M_{{\rm tot}}}\end{equation*}$$

In Equations (1) and (2), ${X_{{\rm tot}}}$ and ${M_{{\rm tot}}}$ are the total concentrations of the ligand and macromolecule, respectively, in the calorimetric cell after *i*th injection and X is the free ligand concentration. Nonlinear least-squares fitting of the data was performed using the same software. The conversion of integral heats ($Q_i^{{\rm tot}{\rm }}$) to differential heats (heats per injection observed in the experiment) and the fitting routine including corrections for heat displacement effects and ligand and macromolecule dilutions in the calorimetric cell were performed as described (69).

The data in Figures 3, 4A and B were fit to Equation (3) to determine $\Delta {C_p}$ (64).

(3) $$\begin{equation*}{\rm{\Delta }}{H_{{\rm obs}}} = \Delta {H_{{\rm obs},\ {\rm ref}}} - \Delta {C_p}(T - {T_{{\rm ref}}})\end{equation*}$$

### Sedimentation velocity

Sedimentation velocity experiments were performed with an Optima XL-A analytical ultracentrifuge and An50Ti rotor (Beckman Coulter, Fullerton, CA, USA) at 42 000 rpm (25°C) as described (50). The concentrations used were 1–2 μM SIPs, 0.3–0.5 μM SSB tetramers, while monitoring absorbance at 230 or 280 nm. The experiments with SSB-Ct peptides were performed in Buffer BTP at pH 8.0 and 50 mM NaCl. The experiment with SSB proteins and the deletion constructs were performed in Buffer BTP at pH 7.0 and 200 mm NaCl. The sample (380 μl) and buffer (394 μl) were loaded into each sector of an Epon charcoal-filled two-sector centerpiece. Absorbance data were collected by scanning the sample cells at intervals of 0.003 cm. Data were analyzed using SEDFIT, to obtain c(s) distributions (70). The c(s) distribution function defines the populations of species with different sedimentation rates and represents a variant of the distribution of Lamm equation solutions (70). The density and viscosity of the experimental buffers at 25°C were determined using SEDNTERP (71). The partial specific volume of each protein used was also determined using SEDNTERP and is as follows: 0.743 ml/g for RecO, 0.742 ml/g for PriA, 0.733 ml/g for χ, 0.735 ml/g for PriC, 0.719 ml/g for wtSSB, 0.722 ml/g for SSBΔ151–166, 0.727 ml/g for SSBΔ130–166, 0.728 ml/g for SSBΔ120–166, 0.704 ml/g for P15, 0.701 ml/g for P31 and 0.694 ml/g for P65. The partial specific volume of (dT)_70_ used was 0.56 ml/g. In the experiments involving more than one species, the partial specific volumes of complexes were calculated assuming additivity using Equation (4), where $n_i$ = number of moles of species ‘i’, $m_i$= molecular weight of species ‘i’, and $\overline{\upsilon_i}$ = partial specific volume of each species i.

(4) $$\begin{equation*}\overline{\upsilon} = \frac{{\mathop \sum \nolimits_i {n_i}{m_i}{{\overline{\upsilon_i}}}}}{{\mathop \sum \nolimits_i {n_i}{m_i}}}\end{equation*}$$

## RESULTS

### SSB-Ct displays binding specificity to RecO, PriA, PriC and χ

It has previously been shown that a peptide, P9, containing only the nine C-terminal residues of SSB (MDFDDDIPF), binds with specificity to two SIPs, PriA and the χ subunit of DNA Pol III (64). In the same study, a peptide containing only the last 15 residues, P15 (Figure 1C), was shown to bind with the same affinity as P9. Here we examined binding of P15 to PriA and χ as well as two additional SIPs, *E. coli* RecO and PriC using ITC. All binding reactions were performed under identical solution conditions, although additional experiments were performed at a lower [NaCl] for PriC as indicated, since the binding affinity was too low to measure at the higher salt concentration.

**Figure 2.**
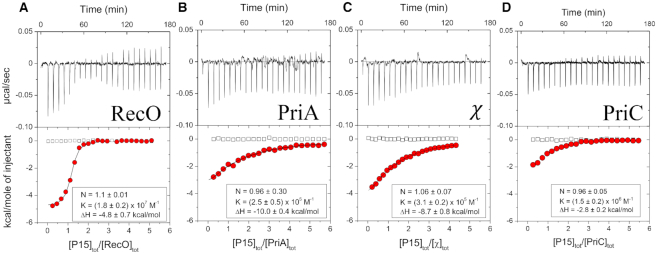
The acidic tip peptide, P15, binds with specificity to four SIPs. The results of ITC titrations of P15 peptide into four SIPs at 25°C, (**A**) RecO (**B**) PriA (**C**) χ in buffer BTP (pH 8.0, 50 mM NaCl) and (**D**) PriC in buffer BTP (pH 8.0, 10 mM NaCl). A titration of P15 with PriC at 50 mM NaCl showed no binding. Upper panels show the raw titration data, plotted as the heat signal (microcalories per second) versus time (minutes), obtained for 22 injections (12 μl each) of P15 (50 μM) into a SIP solution (1–2 μM). Lower panels show the integrated heat responses per injection, normalized to the moles of injected P15, after subtraction of the heats of dilution obtained from the blank titration of P15 into buffer (empty squares). The smooth curves represent the best fit of the data to an n-independent and identical site model. Binding parameters from the fits are indicated in each panel as well as in Table 1 and Supplementary Table S1 (N = stoichiometry, K = association equilibrium binding constant, ΔH = binding enthalpy).

Figure 2, Table 1 and Supplementary Table S1 show the results of ITC experiments examining the binding of P15 to RecO, PriA, and PriC, and χ in buffer BTP plus 50 mM NaCl at 25°C. The best fit results obtained by floating K, N and ΔH are given in Supplementary Table S1, whereas Table 1 shows the best fit binding parameters from the same set of experiments, but constraining the binding stoichiometry to 1.0. P15 binds to each of the four SIPs with a 1:1 stoichiometry, but the equilibrium binding constants differ by two orders of magnitude and display significant differences in binding enthalpy (ΔH). The titrations for RecO, PriA and χ were performed at 50 mM NaCl. However, the binding of P15 to *E. coli* PriC could not be detected at 50 mM NaCl and was therefore examined at 10 mM NaCl to increase the affinity. At 50 mM NaCl, *E. coli* RecO binds with the highest affinity (*K* = (1.2 ± 0.3) × 10^7^ M^−1^), followed by PriA (*K* = (2.6 ± 0.1) × 10^5^ M^−1^) and the χ subunit (*K* = (3.0 ± 0.1) × 10^5^ M^−1^) with comparable values, and then PriC (undetectable). At 10 mM NaCl, PriC binds P15 with *K* = (1.6 ± 0.1) × 10^6^ M^−1^.

**Table 1.** ITC binding data for interaction of *E*. *coli* SSB C-terminal peptides and SIPs

		Stoichiometry (N)	K ($M^{-1}$)	ΔH (kcal/mol)
**RecO**	P15	1 (fixed)	(1.2 ± 0.3) × $10^7$	−5.2 ± 0.1
	P31		(4.1 ± 0.5) × $10^7$	−5.1 ± 0.1
	P65		(3.9 ± 0.8) × $10^7$	−4.7 ± 0.1
**PriA**	P15		(2.6 ± 0.1) × $10^5$	−9.6 ± 0.3
	P31		(4.1 ± 0.2) × $10^5$	−9.7 ± 0.2
	P65		(4.5 ± 0.1) × $10^5$	−9.8 ± 0.1
**DNA Pol III (χ)**	P15		(3.0 ± 0.1) × $10^5$	−9.3 ± 0.1
	P31		(2.8 ± 0.1) × $10^5$	−9.1 ± 0.1
	P65		(7.2 ± 0.4) × $10^5$	−9.3 ± 0.2
**PriC (10 mM NaCl)**	P15		(1.6 ± 0.1) × $10^6$	−2.7 ± 0.1
	P31		(1.3 ± 0.1) × $10^6$	−2.3 ± 0.1
	P65		(8.9 ± 0.9) × $10^5$	−2.6 ± 0.1

K = observed association equilibrium constant, ΔH = enthalpy change.

We performed sedimentation velocity experiments under the same solution conditions used in the ITC experiments to examine the assembly states of each SIP before and after P15 binding with RecO, PriC and χ at 2 μM and PriA at 1 μM with 30-fold excess P15. The sedimentation coefficients were converted to s_20,w_ in order to better compare the experiments at the two different [NaCl]. RecO, PriA and χ are monomeric before and after P15 binding (Supplementary Figure S1). The major PriC species is monomeric under these conditions (48.1%), but shows some higher order species (Supplementary Figure S1c). Binding of P15 favors the PriC monomer and suppresses the higher order species.

### Acidic tip peptides containing additional regions of the IDL show no additional contributions to SIP binding

**Figure 3.**
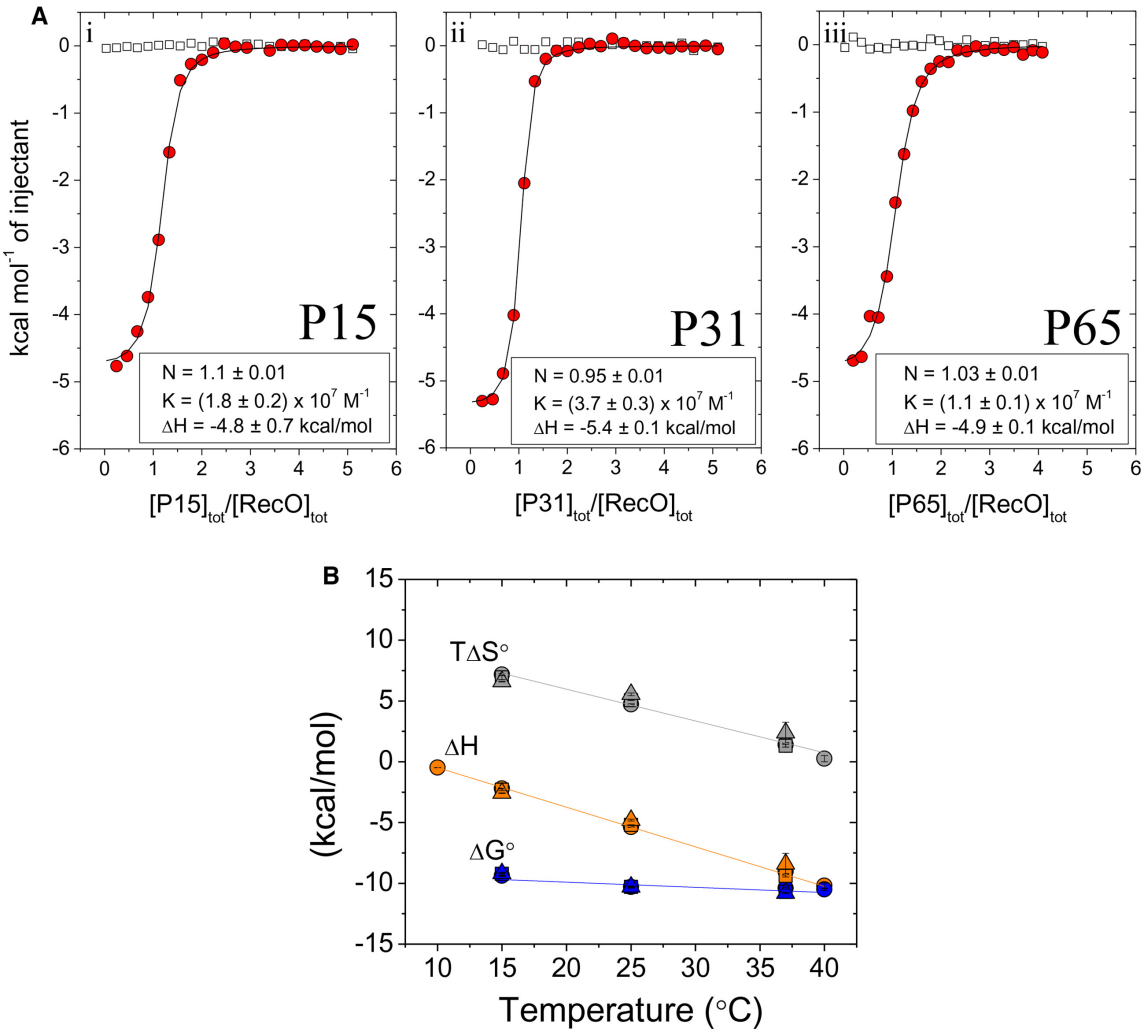
RecO binds to the IDL peptides only via the acidic tip. (**A**) Panels i–iii show the results of ITC studies of the binding of P15, P31 and P65 peptides to RecO, respectively. The peptides (40–50 μM) were titrated into RecO (2 μM) in buffer BTP (pH 8.0, 50 mM NaCl) at 25°C. The smooth curves are simulations for a 1:1 binding model using the best fit binding parameters indicated in each panel (see also Table 1 and Supplementary Table S1). (**B**) Values of ΔG° (blue), ΔH (orange) and TΔS° (gray) obtained from ITC experiments for P15 (○), P31 (Δ) and P65 (□) peptides binding to RecO performed at the indicated temperatures. Solid lines show fits of the data with linear regression. The raw data are shown in Supplementary Figure S3.

A recent study (56) proposed that regions of the intrinsically disordered SSB linkers and not the acidic tips are responsible for SSB binding to some SIPs, including RecO and RecG. In particular, it was suggested that three motifs in the SSB IDL that have proline residues in the first and the fourth positions, PAAP, PQQP and PQQS, are involved in SIP interactions (56). We therefore examined two longer peptides that contain the acidic tip, but also the full IDL (P65) or an additional 22 amino acids of the IDL (P31) to probe for additional interactions with the SIPs (Figure 1C). P15 does not include any of the proline motifs. P31 includes two of the PXXP motifs, and P65, includes all three PXXP motifs. Figure 3A shows the results of ITC experiments examining the binding of each peptide to RecO in buffer BTP plus 50 mM NaCl at 25°C. Within our uncertainties, all three peptides show identical binding affinities and enthalpies, and thus also entropy changes, ΔS°.

To further probe for differences in binding of the three peptides to RecO, we performed titrations at three additional temperatures. The resulting values for ΔG°, ΔH and TΔS° are plotted in Figure 3B and indicate that the full thermodynamic profiles for RecO binding to P15, P31 and P65 are identical. This rules out any fortuitous enthalpy–entropy compensation at any of these temperatures. These results indicate that there are no detectable additional interactions with RecO contributed by the IDL and that the acidic tip is the sole region contributing to binding of RecO. From the linear dependences of ΔH on temperature shown in Figure 3B, we estimate a negative heat capacity change, ΔC_P_ = −326 ± 3 cal/mol⋅deg associated with RecO binding to the acidic tip.

We also performed ITC experiments to examine the binding of P15, P31 and P65 peptides to PriA, PriC and the χ subunit, and the best fit results obtained by floating K, N and ΔH are given in Supplementary Table S1. Table 1 shows the best fit binding parameters from the same set of experiments, but constraining the binding stoichiometry to 1.0. We note that the uncertainties shown in Table 1 and Supplementary Table S1 (±0.1–0.30 kcal/mole in ΔH) were obtained from fits of individual titrations. Supplementary Table S2 compares the binding parameters obtained from fits of replicate experiments. Supplementary Table S2 shows that the uncertainties estimated from repeats of these experiments are slightly larger (±0.5 kcal/mole in ΔH) and encompass the small differences shown in Table 1 and Supplementary Table S1. As with RecO, the binding parameters for the three peptides are the same within error for each individual SIP, indicating no additional contributions to binding from the IDL. However, the binding specificity for the four SIPs observed for P15 in Figure 2 is maintained with P31 and P65.

### RecO binding to full length SSB tetramer

**Figure 4.**
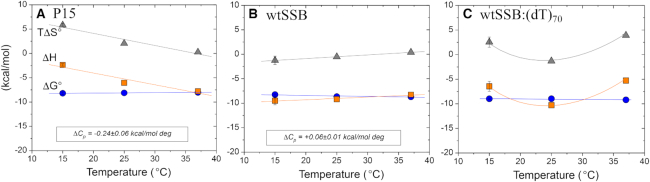
RecO binding to P15, wtSSB and wtSSB–(dT)_70_ indicates that RecO interacts with more than the acidic tip. Thermodynamic parameters for RecO binding to (A)-P15 peptide, (B)-wtSSB per C-terminus and (C)-wtSSB–(dT)_70_ complex per C-terminus in buffer BTP (pH 7.0, 200 mM NaCl); *ΔH*_obs_ (red squares), *ΔG°*_obs_ (blue circles) and *TΔS°*_obs_ (green triangles).

We next examined RecO binding to the full SSB tetramer with its four C-terminal tails. These ITC experiments were performed in buffer BTP at pH 7.0 and 200 mM NaCl to compare with previous studies (64). We could not use 50 mM NaCl due to the low solubility of SSB at this salt concentration. Therefore, we first re-examined RecO binding to P15 in buffer BTP at pH 7.0 and 200 mM NaCl to compare with the RecO-SSB results. The P15-RecO results at three temperatures are shown in Figure 4A, Table 2 and Supplementary Table S3. The equilibrium constant at 200 mM NaCl, pH 8 is lower by a factor of 4 compared to 50 mM NaCl with ∼1.3 kcal/mol change in ΔH. A change in pH from 8.0 to 7.0 at 200 mM NaCl, 25°C, shows a 3-fold reduction in affinity with ∼2 kcal/mol change in binding enthalpy.

**Table 2.** ITC binding data for interaction of *E*. *coli* SSB C-terminal P15 and RecO in buffer BTP

pH	[NaCl] (mM)	Temp.	Stoichiometry (N)	K ($M^{-1}$)	ΔH (kcal/mol)	ΔG° (kcal/mol)	TΔS° (kcal/mol)
8	50	25°C	1 (fixed)	(1.2 ± 0.3) × $10^7$	−5.2 ± 0.1	−9.7 ± 0.2	4.5 ± 0.2
8	200			(3.1 ± 0.3) × $10^6$	−3.9 ± 0.1	−8.9 ± 0.1	5.0 ± 0.1
7	200	15°C		(1.9 ± 0.5) × $10^6$	−2.4 ± 0.2	−8.3 ± 0.2	5.9 ± 0.3
		25°C		(1.1 ± 0.1) × $10^6$	−6.0 ± 0.2	−8.1 ± 0.2	2.0 ± 0.3
		37°C		(4.5 ± 1.1) × $10^5$	−7.8 ± 0.8	−8.1 ± 0.1	0.13 ± 0.1

K = observed association equilibrium constant, ΔH = enthalpy change.

The results of ITC experiments for RecO binding to SSB tetramer in buffer BTP at pH 7.0 and 200 mM NaCl at three temperatures are shown in Figure 4B and the resulting best-fit parameters in Table 3 (N constrained to 4) and Supplementary Table S4 (N floated). At each temperature, the binding stoichiometry, when floated as a parameter, is close to 4 (3.4–4.0) (Supplementary Table S4) indicating that all four SSB C-terminal tails can bind RecO. Interestingly, at 37°C, the thermodynamic profiles for RecO binding to P15 versus SSB (per tail) are identical within our uncertainties suggesting that only the acidic tip contributes to the binding interaction. However, the profiles differ at the lower temperatures of 25°C and 15°C as shown in Figure 4B. The equilibrium binding constant and hence ΔG° are essentially the same for P15 and SSB at 25°C and 15°C. In fact, the ΔG° shows very little temperature dependence for RecO binding to either P15 or SSB. However, ΔH and TΔS° clearly differ. At 15°C, ΔH for RecO binding to P15 is −2.4 ± 0.2 kcal/mole (Table 2), whereas ΔH = −9.8 ± 0.3 kcal/mole for RecO binding to each SSB tail (Table 3). However, the temperature dependent changes in ΔH are nearly entirely compensated by balancing changes in the TΔS° terms, with ΔS° for RecO binding to P15 becoming more favorable at the lower temperatures, whereas the ΔS° term for RecO binding to the SSB tail becomes more unfavorable. In fact, whereas RecO binding to P15 shows a negative heat capacity change (ΔC_p_ = −0.24 ± 0.06 kcal/mol⋅deg), the ΔC_p_ for RecO binding to each SSB tail is slightly positive (ΔC_p_ = 0.06 ± 0.01 kcal/mol⋅deg).

**Table 3.** ITC binding data for interaction of *E*. *coli* SSB constructs and RecO

		Temp.	Stoichiometry (N)	K ($M^{-1}$)	ΔH* (kcal/mol)	ΔG° (kcal/mol)	TΔS° (kcal/mol)
**wtSSB**	apo	15°C	4 (fixed)	(1.2 ± 0.1) × $10^6$	−9.8 ± 0.3	−8.0 ± 0.1	−1.8 ± 0.3
		25°C		(1.5 ± 0.2) × $10^6$	−9.4 ± 0.5	−8.4 ± 0.8	−1.0 ± 0.5
		37°C		(1.3 ± 0.3) × $10^6$	−8.3 ± 0.8	−8.7 ± 0.1	0.4 ± 0.8
	(dT)$_{70}$	15°C		(7.4 ± 1.3) × $10^6$	−6.4 ± 0.2	−9.1 ± 0.1	2.7 ± 0.2
		25°C		(3.8 ± 0.3) × $10^6$	−10.3 ± 0.2	−9.0 ± 0.1	−1.3 ± 0.2
		37°C		(3.0 ± 1.2) × $10^6$	−5.3 ± 0.6	−9.2 ± 0.3	3.9 ± 0.6
**SSBΔ151-166**	apo	25°C		(3.2 ± 0.2) × $10^6$	−7.0 ± 0.1	−8.9 ± 0.1	1.9 ± 0.1
	(dT)$_{70}$	25°C		(3.8 ± 0.2) ×$10^6$	−8.7 ± 0.1	−9.0 ± 0.1	0.3 ± 0.1
**SSBΔ130-166**	apo	25°C		(1.6 ± 0.1) × $10^6$	−2.3 ± 0.1	−8.5 ± 0.1	6.2 ± 0.1
	(dT)$_{70}$	25°C		(5.3 ± 0.5) × $10^6$	−4.5 ± 0.1	−9.2 ± 0.1	4.7 ± 0.1
**SSBΔ120-166**	apo	10°C		(3.8 ± 0.3) × $10^6$	−8.1 ± 0.1	−8.5 ± 0.1	0.4 ± 0.1
	apo	25°C		Not detected	∼ 0		
	(dT)$_{70}$	25°C		(7.0 ± 0.5) × $10^6$	−6.0 ± 0.1	−9.3 ± 0.1	3.3 ± 0.1

K = observed association equilibrium constant, ΔH* = ΔH/4; ΔH per tip.

One caveat with the interpretation of the SSB-RecO binding studies is that there is evidence that the acidic tip can transiently bind to an unoccupied DNA binding domain of SSB with ΔH < 0 (64,72–74). As a result, in order for the acidic tip to bind to RecO, those transient interactions would have to be reversed resulting in a positive contribution to the overall binding ΔH. This was observed previously for SSB binding to the χ subunit of DNA pol III (64). This competitive binding of the acidic tip would result in a less favorable apparent ΔH for SSB binding to RecO (i.e. the observed ΔH of ∼ −10 kcal/mole should actually be more negative in the absence of competitive binding of the tip). However, correcting for this effect makes the difference in ΔH values for P15 versus SSB even greater. Hence, as discussed below, these profiles suggest that RecO interacts with more than just the acidic tip at these lower temperatures.

### RecO binding to SSB in complex with (dT)_70_

We next examined RecO binding to the wtSSB tetramer in complex with (dT)_70_ in buffer BTP at pH 7.0 and 200 mM NaCl to compare with P15 and wtSSB alone. At 200 mM NaCl, SSB forms a 1:1 complex with (dT)_70_ in which all four subunits of the tetramer interact with the DNA (75–77) (see Supplementary Figure S7f). As such, the acidic tips of the C-terminal tails should not bind to the DNA binding domains since these are occupied by (dT)_70_. The results of ITC experiments performed at 15°C, 25°C and 37°C are shown in Figure 4C and Table 3 (*n* = 4 fixed) and Supplementary Table S4 (N floated). At each temperature, the floated binding stoichiometry is near four (Supplementary Table S4) indicating that all four C-terminal tails can bind RecO. Again, the binding affinities (ΔG°) are very similar for RecO binding to SSB–(dT)_70_ versus SSB versus P15 at all temperatures (Table 3). However, the contributions to ΔH and TΔS° differ significantly. Interestingly, the ΔH and TΔS° values for RecO binding to SSB–(dT)_70_ show a non-linear dependence on temperature with a minimum at 25°C indicating that the heat capacity change, ΔC_p,_ is itself temperature dependent, going from negative between 15°C and 25°C to positive between 25°C and 37°C (Figure 4C). These more complicated thermodynamic profiles also suggest that RecO is interacting with more than just the acidic tip.

One caveat in interpreting these experiments is that RecO also has ssDNA binding activity and thus contributions from RecO–(dT)_70_ interactions might be contributing to the overall thermodynamics of binding. However, binding of RecO to part of (dT)_70_ would be expected to make an additional negative contribution to the overall ΔH. This could explain the differences in the ΔH for RecO binding to SSB versus SSB–(dT)_70_ at 15°C and 25°C, but not at 37°C. To examine further whether RecO might bind to the ssDNA in an SSB–(dT)_70_ complex, we performed a control experiment using an SSB variant, SSB-A. SSB-A has an additional 6 amino acids, TGASGT, extending from the C-termini of wtSSB that eliminates binding to RecO (Supplementary Figure S7a and b), yet maintains DNA binding activity similar to wtSSB (Supplementary Figure S7e and f). RecO shows no binding to an SSB-A:(dT)_70_ complex at 25°C (Supplementary Figure S7b), indicating that the observed binding of RecO to wtSSB–(dT)_70_ complex requires a canonical acidic tip. This experiment also indicates that any interactions of SSB with RecO outside of the acidic tip are too weak to be observed in the absence of the acidic tip.

### Deleting part or all of the IDL affects RecO binding to SSB

**Figure 5.**
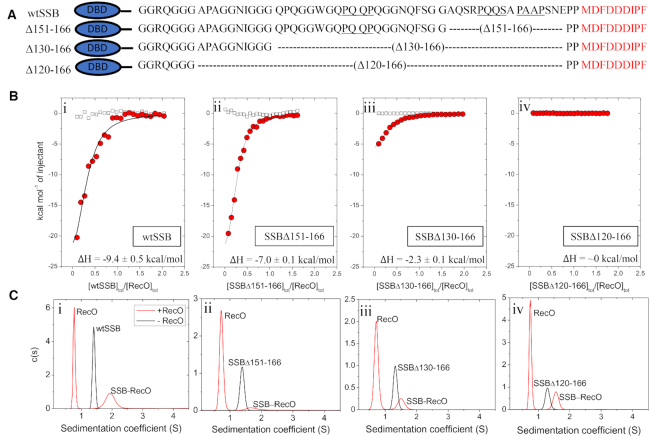
RecO binding to SSB tetramers with portions of the IDL deleted. (**A**) Schematics and sequences of the series of SSB variant tetramers with varying C-terminal IDL deletions, where the PXXP motifs are underlined. The amino acid residues deleted in each variant are denoted. The wtSSB DNA binding domain (DBD) is shown as an ellipse (blue). (**B**) Results of ITC experiments for RecO binding to tetramers of: (i)-wtSSB, (ii)-SSBΔ151-166, (iii)-SSBΔ130-166 and (iv)-SSBΔ120-166. SSB constructs (9–10 μM) were titrated into RecO (1 μM) in buffer BTP (pH 7.0, 200 mM NaCl) at 25°C. The smooth curves are simulations of Equations (1) and (2) using the best fit parameters determined from each titration. The ΔH for RecO binding per C-terminus is indicated on each panel. The binding parameters are shown in Table 3 and Supplementary Table S4. (**C**) The results of sedimentation velocity experiments, plotted as the c(s) distribution (70) for each SSB construct (0.35 μM tetramer) in the absence (black lines) and presence (red lines) of 2.5 molar excess of RecO (3.5 μM) per C-terminus. The sedimentation coefficients of each SSB are 1.4 S, 1.4 S, 1.3 S and 1.3 S, respectively, corresponding to the tetrameric species. Two peaks are observed in the presence of RecO. The peak at 0.8 S is due to free RecO. The SSB peaks are shifted to 1.7 S, 1.6 S and 1.5 S, and 1.6S, respectively, indicating binding of at least one RecO per SSB tetramer.

We next compared RecO binding to wtSSB tetramer and SSB tetramers containing different IDL deletions, SSBΔ151-166, SSBΔ130-166 and SSBΔ120-166, all of which still retain the nine-residue acidic tip as shown in Figure 5A. These constructs have been described and characterized previously and all form stable tetramers under the conditions of our experiments (49). The SSBΔ151-166 construct has the two C-terminal PXXP motifs (PAAP and PQQS) deleted, whereas SSBΔ130-166 and SSBΔ120-166 have all three PXXP motifs deleted. ITC experiments were performed in buffer BTP (pH 7) with 200 mM NaCl at 25°C in order to increase protein solubility and the results are shown in Figure 5B, Table 3 and Supplementary Table S4. The first observation is that under the same conditions at 25°C, RecO binding to wtSSB (*K* = (1.5 ± 0.2) × 10^6^ M^−1^, ΔH = −9.4 ± 0.5 kcal/mol; Table 3) is slightly more favorable than to P15, P31 and P65 (*K* = (1.1 ± 0.1) × 10^6^ M^−1^, ΔH = −6.0 ± 0.2 kcal/mol; Table 2). The fact that the ΔH is more favorable for wtSSB binding suggests the presence of additional interactions with RecO in addition to the acidic tip. Interestingly, although the binding affinities of RecO for wtSSB and the two variants, SSBΔ151-166 and SSBΔ130-166, show only small differences at 25°C, ΔH becomes less favorable as more of the linker is deleted, with ΔH changing from −9.4 ± 0.5 kcal/mol, to −7.0 ± 0.1, and −2.3 ± 0.1 for wtSSB, SSBΔ151-166 and SSBΔ130-166, respectively. In fact, binding of SSBΔ120-166 to RecO is undetectable by ITC under these conditions (Figure 5Biv) (indicating either that ΔH ∼0 at 25°C or that this mutant does not bind to RecO).

In order to determine if the inability to detect binding of RecO to SSBΔ120-166 by ITC is due to a zero ΔH or to a loss of RecO binding, we examined binding to SSBΔ120-166 by sedimentation velocity. The results, plotted as c(s) distributions (70), are shown in Figure 5Ci–iv. The SSB variants alone (black lines) show single peaks indicating that wtSSB and all three SSB variants are stable tetramers at 0.35 μM. In the presence of a 10-fold molar excess of RecO protein to SSB tetramer, two species are observed in each experiment (Figure 5C, red lines), indicating that RecO forms complexes with wtSSB and each SSB variant. Therefore, the inability to detect binding of RecO to SSBΔ120-166 by ITC is due to a near zero net enthalpy change at 25°C. An additional ITC experiment examining SSBΔ120-166 binding to RecO performed at 10°C (Supplementary Figure S5a) shows detectable binding with *K* = (3.7 ± 0.3) × 10^5^ M^−1^ and ΔH = −8.1 ± 0.1 kcal/mol. This indicates a positive ΔC_p_ (∼ 0.54 kcal/mol⋅deg) for RecO binding to SSBΔ120-166. The expected value of ΔH for P15 binding to RecO at 10°C, extrapolated from the data in Figure 4A, is ∼−1.8 kcal/mol, significantly smaller than ΔH = −8.1 ± 0.1 kcal/mol for RecO binding to SSBΔ120-166. This comparison also suggests that RecO makes additional contacts with SSB in addition to the acidic tip, however, these interactions cannot be with PXXP motifs of the IDL since these are missing in SSBΔ120-166.

### Binding of (dT)_70_ to the DNA binding domain of SSB increases RecO affinity for SSB

**Figure 6.**
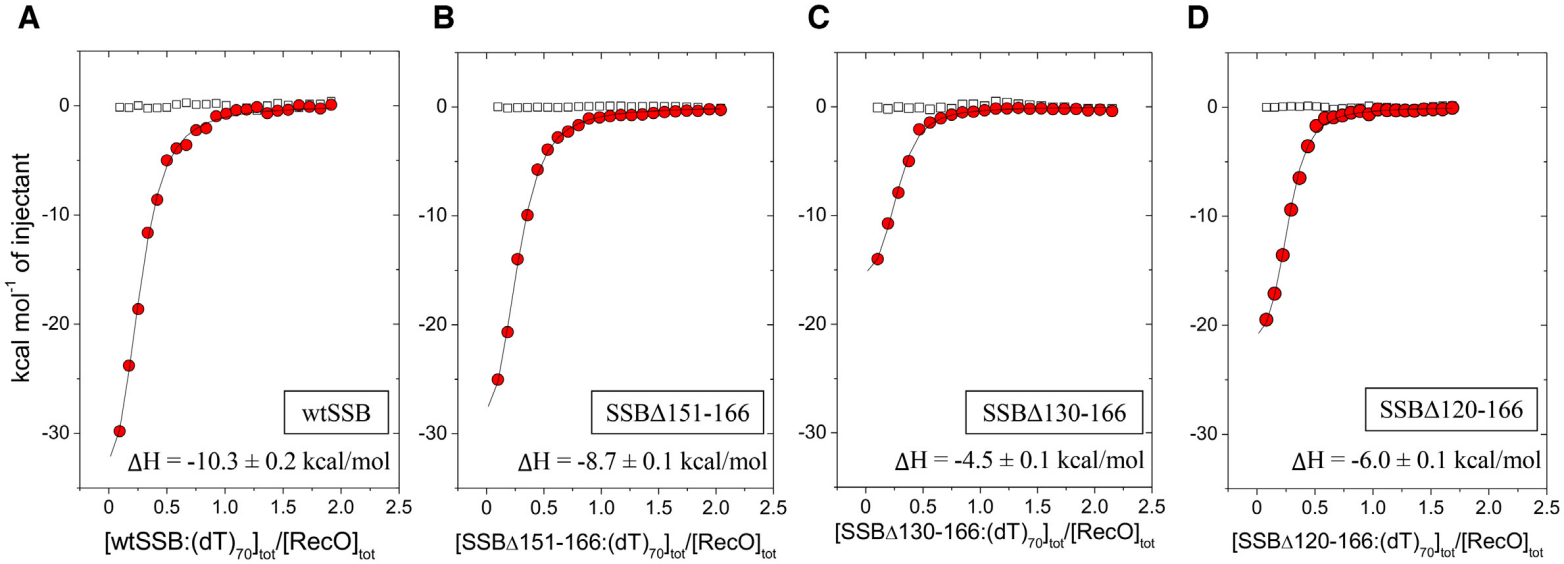
RecO binding to the linker deletion variants of SSB tetramer–(dT)_70_ complexes. Results of ITC experiments for RecO binding to complexes of (dT)_70_ bound to the SSB tetramers: (**A**)-wtSSB, (**B**)-SSBΔ151-166, (**C**)-SSBΔ130-166 and (**D**)-SSBΔ120-166. SSB–(dT)_70_ complexes (9–10 μM) were titrated into RecO (1 μM) in buffer BTP (pH 7.0, 200 mM NaCl) at 25°C. The smooth curves are simulations of Equations (1) and (2) using the best fit parameters determined from each titration. The ΔH for RecO binding per C-terminus is indicated on each panel. The binding parameters are shown in Table 3.

Previous studies of SSB binding to PriA and the χ subunit of DNA pol III (64) as well as DNA binding studies (72), indicate that the acidic tip can interact transiently with any unoccupied ssDNA binding sites (DBDs) on SSB. This, in turn, will result in lower apparent binding affinities of a SIP for the acidic tip when attached to the SSB protein. However, when SSB is bound to (dT)_70_ in its (SSB)_65_ binding mode, all of the ssDNA binding sites are occupied by ssDNA, eliminating the competitive binding of the acidic tip to the DBD (64). We therefore examined RecO binding to wtSSB and the SSB IDL deletion variants in complex with (dT)_70_. SSB and the deletion variants bind (dT)_70_ stoichiometrically in the (SSB)_65_ mode with ssDNA wrapping around all four subunits (49,64). The results of ITC experiments are shown in Figure 6, Table 3 and Supplementary Table S4. When compared to apo wtSSB tetramer, the binding affinity and enthalpy are slightly increased for RecO binding to the wtSSB–(dT)_70_ complex. The SSBΔ130-166 variant shows an even larger increase in RecO binding affinity and enthalpy when bound to (dT)_70_. For the SSBΔ120-166 variant in complex with (dT)_70_, binding is observed with ΔH = −6.0 ± 0.1 kcal/mol. This is in stark contrast to RecO binding to the apo SSBΔ120-166 variant where binding could not be detected by ITC at 25°C due to a net zero ΔH. This could be explained if the acidic tips of the apo SSBΔ120-166 variant interact strongly with the DBD with ΔH = −6 kcal/mol due to increased proximity to the DBD as a result of the much shorter nine amino acid IDL. As such, the acidic tip would need to dissociate from the DBD, resulting in a +6.0 kcal/mol contribution to ΔH that is completely offset by the ΔH for acidic tip binding to RecO (−6.0 kcal/mol) resulting in a net ΔH ∼ 0. A control ITC experiment showed no detectable interaction between the P65 peptide and (dT)_70_ (Supplementary Figure S7d).

## DISCUSSION

*Escherichia coli* SSB protein is an essential protein and a central player in all aspects of genome maintenance. It not only functions to bind and stabilize ssDNA, but also serves as a central hub to bind more than 17 other proteins (SIPs) and bring them to their sites of function. Although many SIPs have been identified, the importance of binding specificity for these SIPs remains to be determined. Important questions include how the SSB–ssDNA binding modes influence SIP interactions and *vice versa*. It is clear that SIPs can influence the relative stability of the SSB–ssDNA binding modes, generally favoring the (SSB)_35_ mode (17,60,65). The intrinsically disordered C-terminal tails of SSB are essential for all SSB–SIP interactions examined so far and the major point of interaction of SIPs with SSB occurs via the nine amino acid acidic tip at the C-termini of the SSB subunits (5,23,64,78–82). However, it is not clear why the acidic tip of *E*. *coli* SSB resides at the C-terminal end of a 56 amino acid IDL. Is the IDL simply a tether needed to enable SIPs to remain bound to SSB but also reach more distant sites on the DNA? It has recently been shown that the SSB IDL is essential for the highly cooperative binding of SSB to ssDNA (49,50). However, there have been recent suggestions that the IDL may also participate in SSB–SIP interactions (55,56).

### SSB shows specificity for SIP binding

The results reported here show that three peptides containing the acidic tip and different amounts of the IDL have identical thermodynamic binding parameters for an individual SIP. However, the binding profiles differ for each SIP indicating specificity for the acidic tip. At 25°C, the binding affinities vary by a factor of 100 with the following ranking from the strongest to the weakest binding: RecO > PriA ∼ χ > PriC (Table 1). The binding enthalpies also range from ∼ −2.7 to −9.6 kcal/mol indicating that the binding specificity is temperature dependent. For SIPs, in which the sites of interaction of the SSB acidic tip have been identified either crystallographically or by nuclear magnetic resonance, such as *E. coli* RecO (19), *Klebsiella pneumoniae* PriA (83), *E. coli* RecQ (15,17), PriC (28,65) and ExoI (32), the sites are generally positively charged and highly conserved, but not identical. The last two C-terminal residues of the tip (Pro and Phe) interact with a hydrophobic pocket in the central alpha helical region of RecO, distinct from the OB-fold, similar to what is observed for ExoI binding (32). Two specific Arg residues have been identified in PriC as interacting with the Asp residues in the tip (49). In PriA, the tip is bound to the junction of the helicase core, a part of the DNA binding domain, and a part of the helicase domain that is on the opposite face of the structure relative to the DNA-binding surface. On the other hand, the acidic tip appears to interact with the winged helix sub-domain of the catalytic core of *E. coli* RecQ, which forms the DNA-binding surface (5). Since the four SIPs show clear specificity for binding to the SSB acidic tip, this will affect their recruitment by SSB during the course of genome maintenance.

### Does the SSB IDL contribute to SSB–SIP interactions?

A recent proposal has been made that the C-terminal acidic tip of SSB is not involved in SSB–SIP interactions, but rather that regions of the IDL provide the major sites of interaction with some SIPs (55,56). Bianco *et al.* (56) suggested that the IDL region that includes three PXXP motifs are involved in binding to two SIPs, RecO and RecG, using SSB linker deletion variants similar to those investigated here. It was suggested that regions of the IDL containing proline might form a polyproline II helix that could interact with the OB-folds of these SIPs. The experiments reported here directly test this hypothesis. The results of our experiments examining the binding of isolated peptides containing the SSB acidic tip and different amounts of the IDL yield unequivocal results. In the absence of the SSB DNA binding domain, the four SIPs, RecO, PriA, PriC and the χ subunit of DNA pol III interact identically with all three peptides. Our experiments with RecO show identical full thermodynamic profiles, ΔG°, ΔH, TΔS° and ΔC_p_, for binding to all three peptides. Since the shortest peptide contains the acidic tip without any PXXP motifs, whereas the longest peptide contains the acidic tip plus the entire 56 amino acid IDL including all three PXXP motifs, these results indicate that only the acidic tip is involved in the binding of these peptides to all four SIPs, with no additional contributions from the IDL.

### Evidence for additional interactions between SSB and RecO

A comparison of the binding of RecO to the acidic tip peptides versus wtSSB and wtSSB–(dT)_70_ complex shows very different thermodynamic profiles. Although there are only small differences in binding affinity (ΔG°) among all three, there are large differences in the enthalpic and entropic contributions. In particular RecO binding to wtSSB shows a much more favorable ΔH and compensating unfavorable TΔS° term, compared with P15 binding. This provides strong evidence that RecO binds to the SSB tetramer via interactions in addition to those made with the acidic tip. The thermodynamic profile for RecO binding to the wtSSB–(dT)_70_ complex is even more complex, showing non-linear dependences of ΔH and ΔS° on temperature indicating a temperature dependent ΔC_P_. These results also suggest additional interactions of RecO with the tetrameric core of SSB, although it is possible that some of these differences reflect interactions of RecO with the DNA. While control experiments with SSB-A, which has six amino acids added to each of the acidic tips, indicates no binding of RecO to an SSB-A–(dT)_70_ complex, RecO–ssDNA interactions could occur if there is an SSB-DNA binding mode change induced by RecO binding to the acidic tip. However, this additional interaction between RecO and SSB does not involve the PXXP motifs in the IDL since RecO can still bind an SSB variant, SSBΔ120-166, even though it contains only nine residues of the IDL, but none of the three PXXP motifs. It is very interesting to note that the ΔG° values for RecO binding to P15, SSB and SSB–(dT)_70_ are nearly temperature independent due to compensating changes in the enthalpic and entropic contributions.

**Figure 7.**
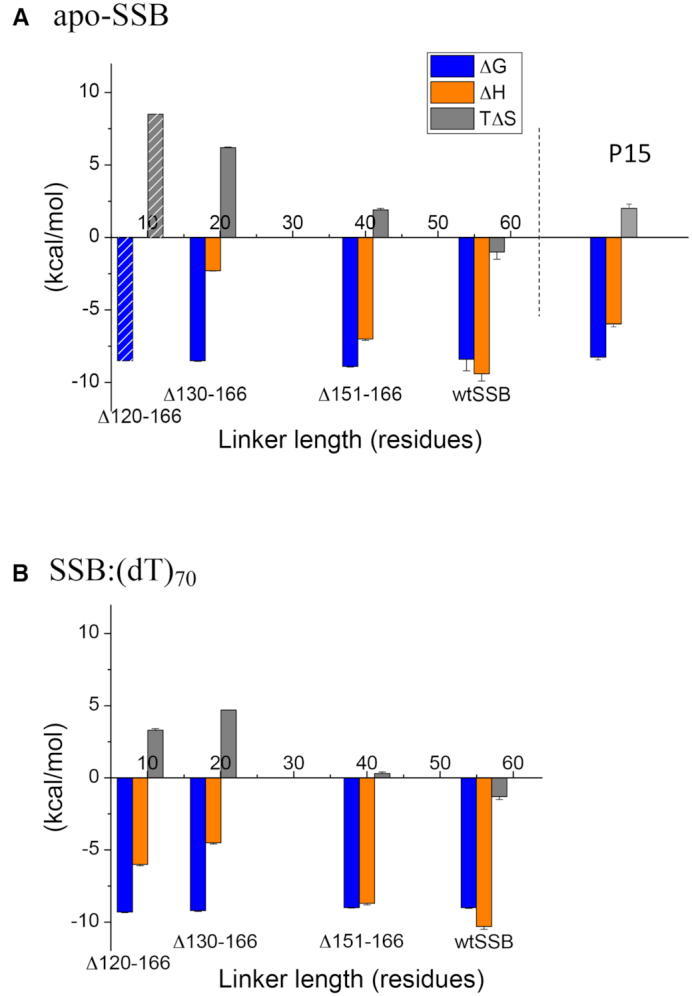
Thermodynamic parameters for the interactions of RecO with wtSSB and three SSB linker deletion variants. The thermodynamic parameters (per SSB C-terminus) obtained from the ITC experiments in Figures 5 and 6 (from Supplementary Table S4) are shown for binding of RecO to wtSSB, SSBΔ151-166, SSBΔ130-166 and SSBΔ120-166. (**A**) Values for RecO binding to apo-SSB tetramers; ΔG° (left-blue), ΔH (middle-orange), TΔS° (right-gray). Since ΔH is undetectable (∼0 kcal/mol) for SSBΔ120-166 binding to RecO, the indicated values of ΔG° and TΔS° were estimated by assuming that ΔG° is the same as for SSBΔ130-166. The parameters obtained for P15 binding to RecO are shown for comparison. (**B**) Values for RecO binding to SSB tetramers bound to (dT)_70_; ΔG° (left-blue), ΔH (middle-orange), TΔS° (right-gray).

Figure 7A compares the thermodynamic profiles at 25°C for RecO Binding to P15, wtSSB and the three SSB linker deletions. As noted above, the affinity, ΔG°, is essentially independent of linker length. However, ΔH, and therefore TΔS°, are clearly affected by shortening the linker length. The favorable ΔH decreases continuously from −9.4 ± 0.5 kcal/mol for wtSSB to ΔH = −2.3 ± 0.1 kcal/mol for SSBΔ130-166 to ΔH ∼0 for SSBΔ120-166. However, this is compensated by an increase in the TΔS° term going from a slightly unfavorable TΔS° = −1.0 ± 0.5 kcal/mol for wtSSB to a very favorable TΔS° = +6.2 ± 0.1 kcal/mol for SSBΔ130-166 (Table 3) to an estimated TΔS° = +8.5 kcal/mol (assuming the same ΔG° = −8.5 kcal/mol as for SSBΔ130-166). The largest favorable ΔS° observed for the SSBΔ120-166 with the shortest linker is consistent with our hypothesis that the transient interaction between the acidic tip and the DBD of SSBΔ120-166 constrains the acidic tip conformationally in the apo-SSBΔ120-166 and that RecO binding relieves this constraint resulting in a favorable ΔS°. On the other hand, the wild-type linker at 56 residues should be conformationally more flexible, resulting in a less favorable contribution to ΔS°. We expect there to be less of a difference in the conformational entropy changes associated with RecO binding to the (dT)_70_ complexes with the SSB linker deletion variants since the transient interactions of the acidic tip with the DNA binding domains of SSB should be eliminated in the SSB–(dT)_70_ complexes. This is qualitatively observed in Figure 7B which shows an overall decrease in the favorable ΔS° for RecO binding to the (dT)_70_-SSB variants compared to the apo-SSB variants.

### The DNA binding domains of SSB compete with SIPs for acidic tip binding

Previously, Kozlov *et al.* (64) provided evidence that in wtSSB, the DBD can interact with the acidic tip and thus compete with PriA and χ for binding of the acidic tip. Our current study comparing RecO binding to wtSSB and three SSB variants with shorter linker lengths provides additional evidence for interactions between the acidic tip and the DBD in the apo SSB variants and that the probability of these interactions increases as the linker length is shortened. The prime evidence for this effect is the continuous decrease in favorable ΔH for RecO binding as the linker length decreases. One explanation for this trend is that the local concentration of the acidic tips in the vicinity of the DBD will increase as the linkers are shortened, promoting tip binding to the DBDs. For example, RecO binding to SSBΔ120-166 shows a net ΔH ∼0, which is consistent with ΔH = −6 kcal/mol for P15 binding to RecO and a compensating ΔH = +6 kcal/mol for the acidic tip dissociating from the DBD.

The competitive binding of the tips to the DBD can be eliminated, or at least reduced, by forming an SSB–DNA complex with (dT)_70_ in the 65 mode so that all four DBDs are occupied by DNA (38). When DNA is bound to the DBD, the acidic tip no longer interacts with the DBD and thus is more available to interact with a SIP. This is supported by the observation that ΔH = −6.0 ± 0.1 kcal/mol for RecO binding to the SSBΔ120-166–(dT)_70_ complex, which should not contain contributions from the acidic tip-DBD intramolecular interactions. This effect has previously been demonstrated for the χ subunit of DNA Pol III (64). Furthermore, the affinity and ΔH for χ binding to the C-terminal nine residues of SSB (P9) are very similar to χ binding to (dT)_70_-bound SSB, indicating that interactions between SSB and χ occur only through the tip.

We note that the analysis of RecO binding to the SSB–(dT)_70_ complex may be complicated by the fact that RecO also has ssDNA binding activity and thus may interact with the DNA that is bound to SSB. For the SSB variants with the shorter linkers, the local concentration of RecO around SSB-bound DNA will increase, and this might facilitate a RecO-induced partial displacement of DNA. Such observations have been reported for PriA and PriC, which facilitate a transition from the (SSB)_65_ mode to the (SSB)_35_ mode upon binding SSB (60,63). It is also possible that some unfavorable steric interactions might occur upon RecO binding to an acidic tip that is too close to the SSB tetramer and thus contribute to differences in the thermodynamic profiles.

Overall, our results with the linker peptides indicate that the SSB acidic tip is solely responsible for interactions with the four SIPs examined here, including RecO, with no contributions from the IDL. However, the binding of RecO to full length SSB as well as the three SSB variants with different linker deletions show complex thermodynamic profiles that further depend on whether the SSB tetramers are bound to ssDNA. It is interesting that the distribution of IDL lengths among bacterial SSB proteins ranges from 16 to 126 amino acids (49). However, the amino acid compositions are similar having few charged residues and being generally rich in glycines and prolines, thus favoring a more collapsed state (49,84). Hence, the binding behaviors of the SSB-linker deletion variants reported in this paper are likely relevant to the binding of SIPs to SSBs from other bacteria. It is interesting in this regard that the profound enthalpy/entropy compensations that we observe for RecO binding to the acidic tip result in binding affinities that are relatively insensitive to the IDL length and whether the acidic tip is attached to SSB or an SSB–ssDNA complex.

## Supplementary Material

gkz606_Supplemental_File
