# Supplementary material for: Are the intrinsically disordered linkers involved in SSB binding to accessory proteins?
Source: Nucleic Acids Res. 2019 Jul 22;47(16):8581–94. doi: 10.1093/nar/gkz606 (PMC7145534; doi:10.1093/nar/gkz606)
Supplement: gkz606_Supplemental_File [file gkz606_supplemental_file.pdf]

SUPPLEMENTARY MATERIAL

Are the Intrinsically Disordered Linkers Involved in SSB Binding to Accessory Proteins?

Min Kyung Shinn[1,2], Alexander G. Kozlov[1], Binh Nguyen[1], Wlodek Bujalowski[3]

and Timothy M. Lohman[1]*

[1]Department of Biochemistry and Biophysics, Washington University in St. Louis School of Medicine, St. Louis, MO 63110

[2]Department of Physics, Washington University in St. Louis, St. Louis, MO 63130

[3]Department of Biochemistry and Molecular Biology, The University of Texas Medical Branch at Galveston, Galveston, TX 77555

*To whom correspondence should be addressed. E-mail: lohman@wustl.edu

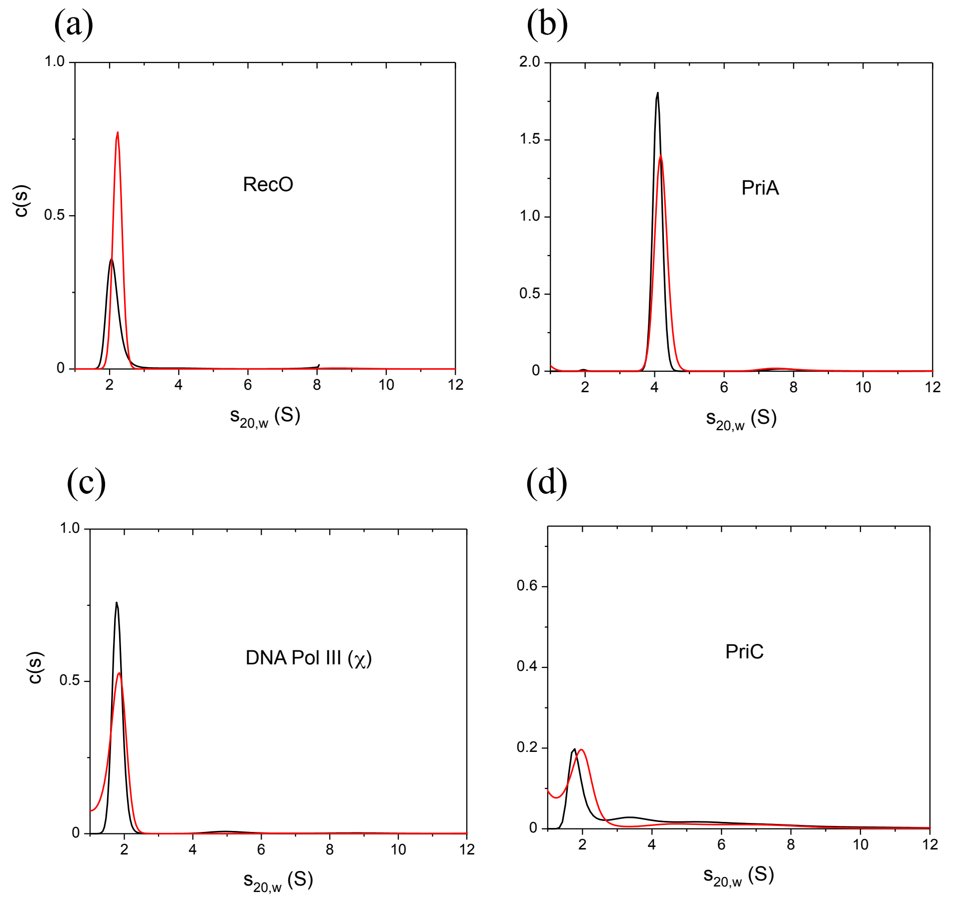

Figure S1. Effect of P15 peptide binding on the assembly state of the four SIPs. (a) RecO (b) PriA (c) χ (d) PriC. The results of sedimentation velocity experiments, plotted as c(s) distributions for each SIP (1 – 2 μM) in the absence (black lines) and presence (red lines) of a 30-fold molar excess of P15 peptide (30 - 60 μM). The sedimentation coefficients were converted to conditions of water at 20°C ($s_{20,w}$) to compare RecO, PriA, and χ at 50 mM NaCl and PriC at 10 mM NaCl in buffer BTP (pH 8.0), solution conditions identical to those used in the ITC experiments shown in Figure 2. The sedimentation coefficients are 1.89 S for PriC, 4.09 S for PriA, 1.76 S for χ, and 2.06 S for RecO indicating these SIPs are monomeric. The peaks are shifted to 1.95 S for PriC, 4.19 S for PriA, and 1.80 S for χ, and 2.16 S for RecO. The shifts in the peaks reflect binding of P15 to the SIPs. In (d), PriC shows a small population of higher order oligomeric species both in the absence and presence of P15. However, the higher order species at ~3.5 S is suppressed in the presence of P15.

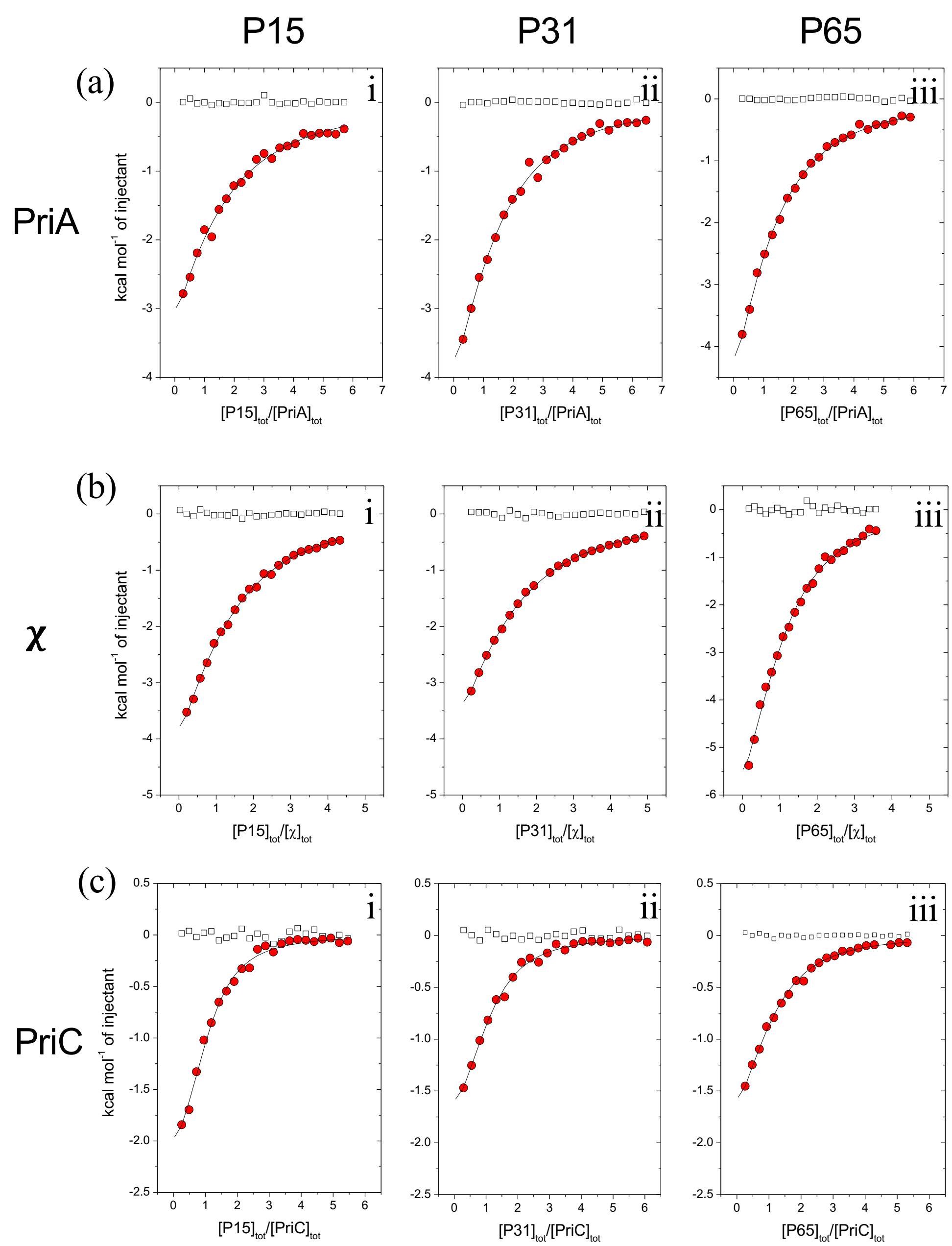

Figure S2. Results of ITC experiments for SIPs binding to P15, P31, and P65 peptides (a) PriA, (b) PriC, and (c) χ. The panels are arranged as follows for each SIP: (i) P15, (ii) P31, and (iii) P65. The SIPs (2 μM) were titrated with peptide (40 – 50 μM) in buffer BTP (pH 8.0, 50 mM (PriA and χ) or 10 mM (PriC)) at 25°C. The fitted binding parameters (N = stoichiometry, K = equilibrium binding constant, and ΔH = binding enthalpy) are given in Tables 1 and S1. The smooth curves are simulations using eqs. (1) and (2) and the best fit parameters determined from each titration.

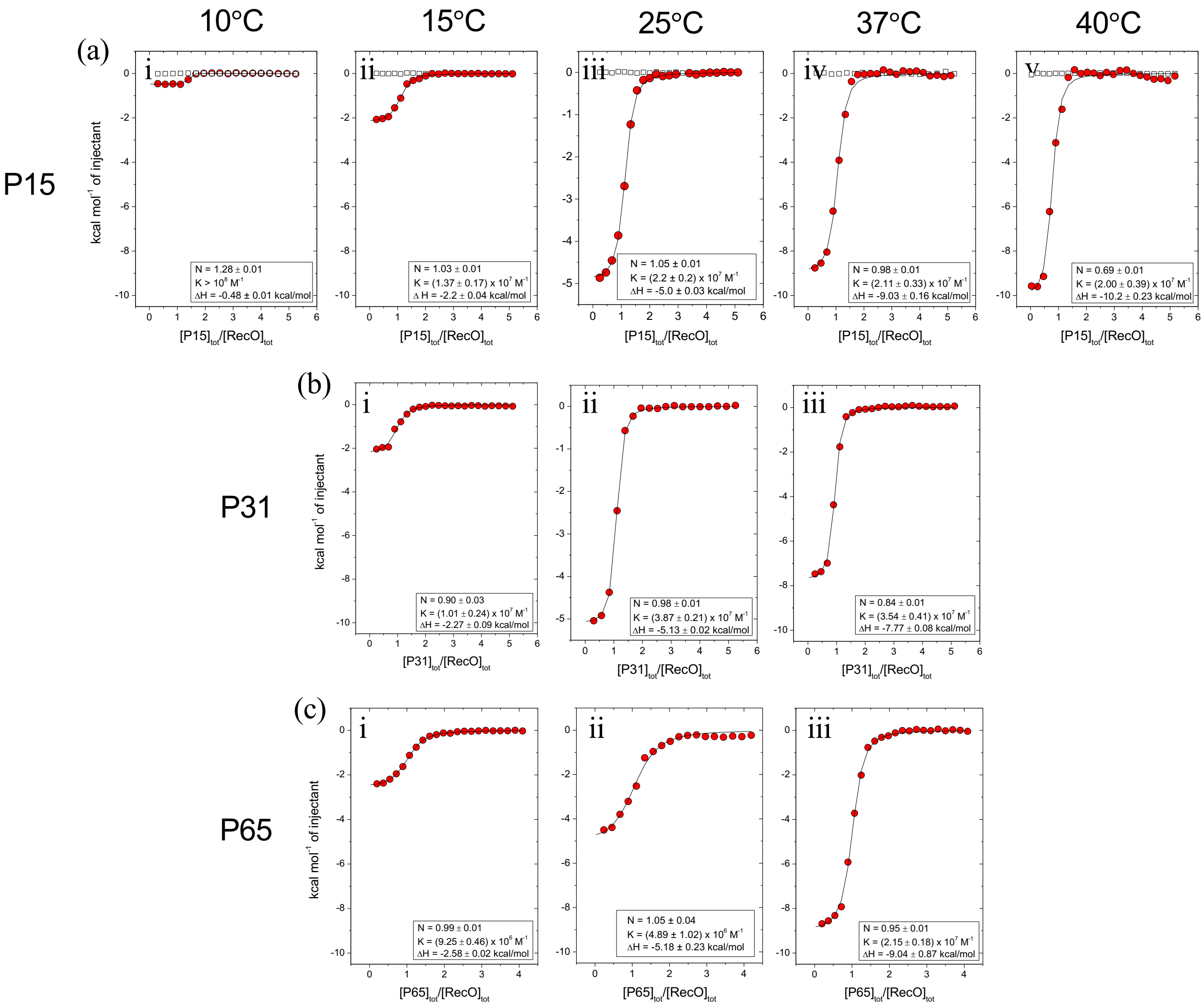

Figure S3. Temperature dependence of SSB-Ct peptides binding to RecO. (a)P15; 10°C, 15°C, 25°C, 37°C, 40°C (b) P31; 15°C, 25°C, 37°C (c) P65; 15°C, 25°C, 37°C. For all ITC experiments, peptides (40 – 50 μM) are titrated into RecO (2 μM) in buffer BTP (pH 8.0, 50 mM) at the indicated temperature. The thermodynamic parameters determined by fitting the ITC titrations (ΔG°, ΔH, and TΔS°) are plotted in Figure 3. The smooth curves are simulations using eqs. (1) and (2) and the best fit parameters determined from each titration.

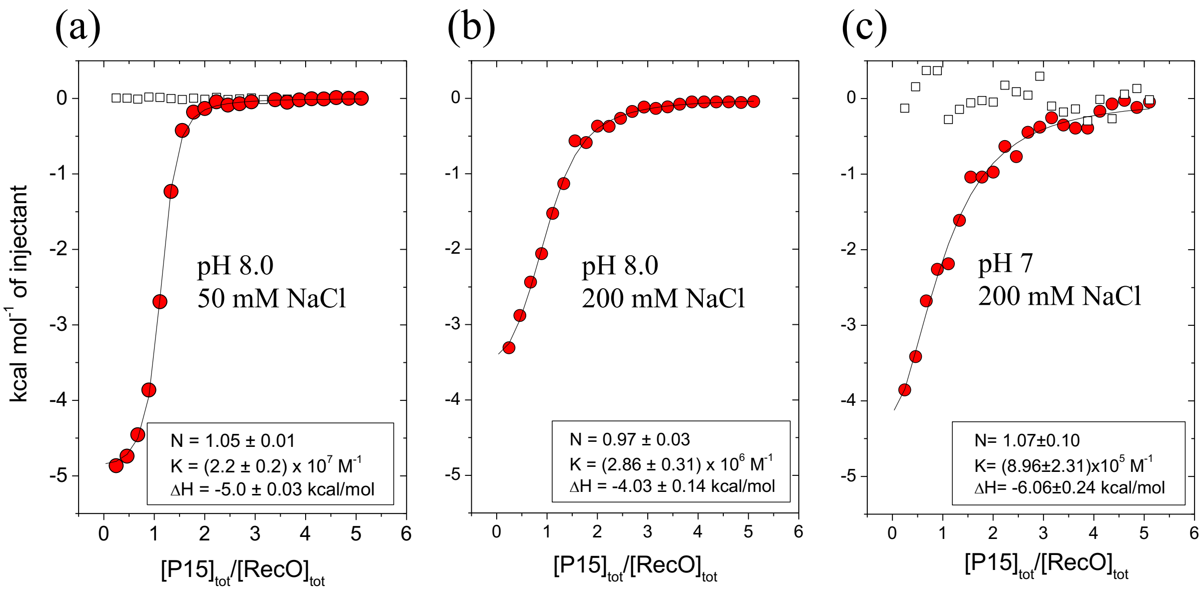

Figure S4. Effects of pH and [NaCl] on SSB-Ct peptide binding to RecO. P15 peptide (50 μM) is titrated into RecO (2 μM) in buffer BTP at the specified pH and [NaCl]. (a) pH 8.0, 50 mM NaCl (b) pH 8.0, 200 mM NaCl (c) pH 7.0, 50 mM NaCl. The best fit binding parameters are given in Tables 2 and S2. The smooth curves are simulations using eqs. (1) and (2) and the best fit parameters determined from each titration.

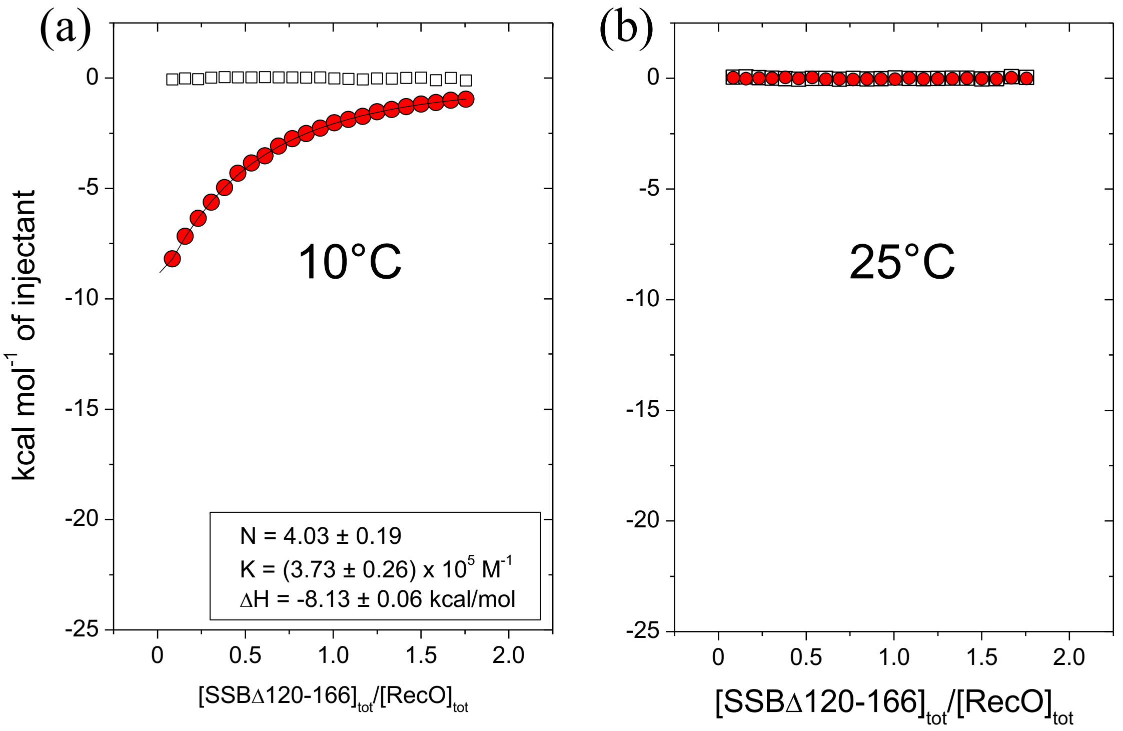

Figure S5. Results of ITC experiments for SSBΔ120-166 binding to RecO at (a) 10°C and (b) 25°C. SSBΔ120-166 (10 μM) was titrated into RecO (1 μM) in buffer BTP (pH 7.0, 200 mM NaCl). The smooth curve in (a) is a simulation using eqs. (1) and (2) and the best fit parameters determined from each titration.

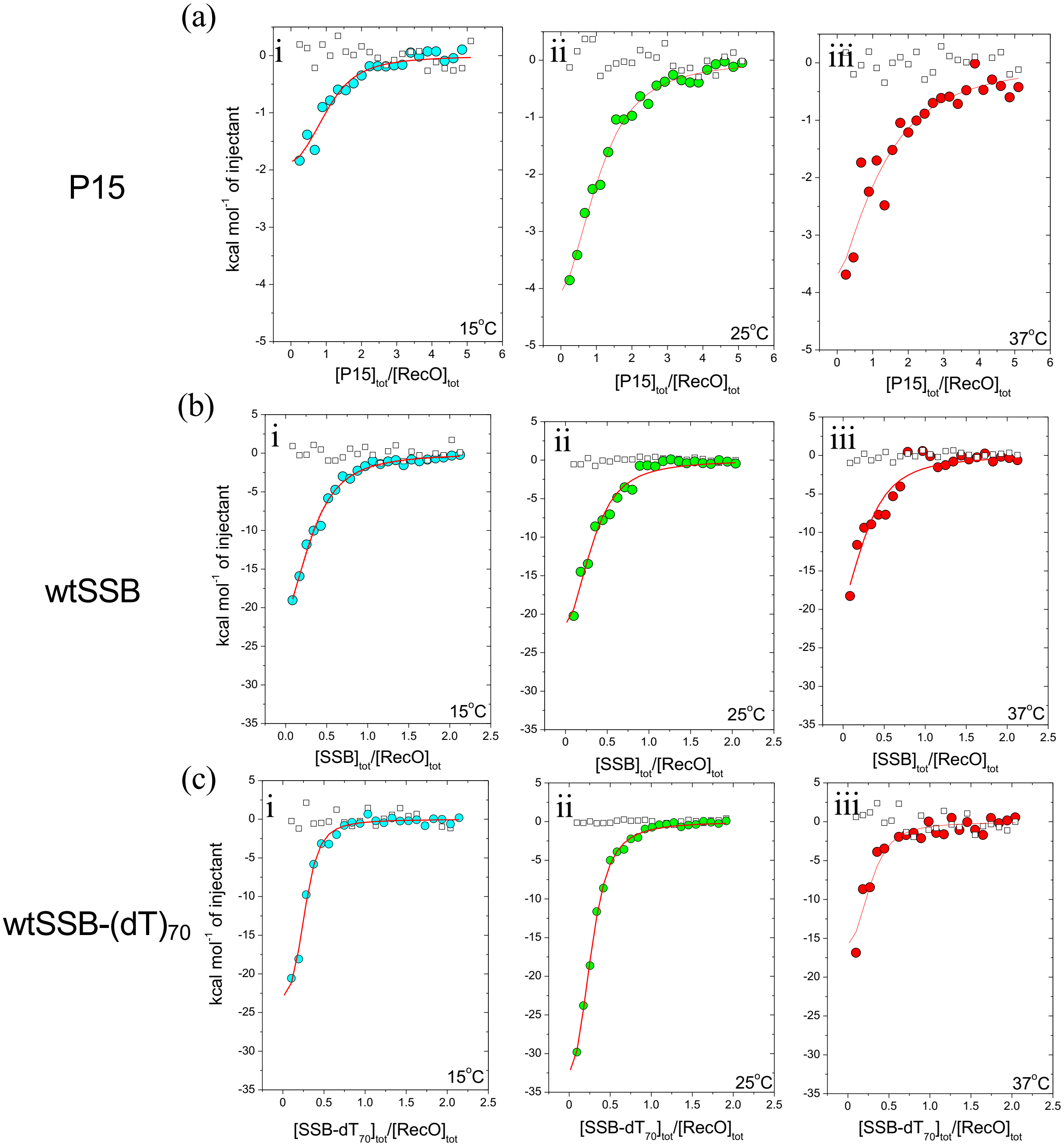

Figure S6. Results of ITC experiments for RecO binding to (a) P15 (b) wtSSB (c) a 1:1 molar complex of wtSSB bound to $(dT)_{70}$ at (i) 15°C (ii) 25°C (iii) 37°C. (a) P15 (50 μM) (b) wtSSB (10 μM) (c) wtSSB pre-bound to equimolar $(dT)_{70}$ (15 μM) were titrated into RecO (1 – 2 μM) in buffer BTP (pH 7.0, 200 mM NaCl) at the indicated temperatures. The fitted binding parameters are given in Tables 2, 3, S2, and S3. The smooth curves are simulations using eqs. (1) and (2) and the best fit parameters determined from each titration.

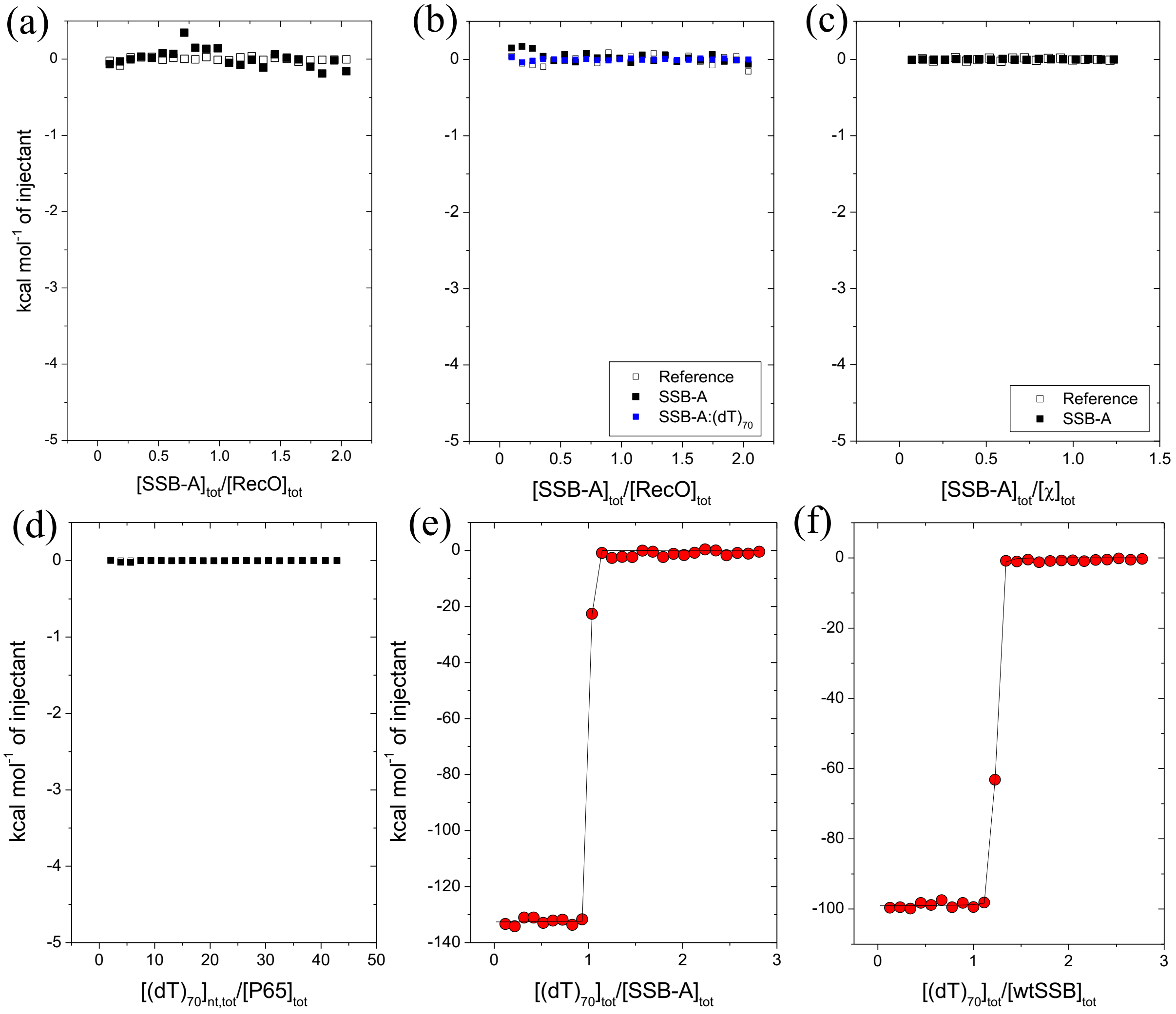

Figure S7. Results from various control ITC titrations of (a) SSB-A and RecO at 15°C and (b) 25°C, (c) SSB-A and χ, (d) P65 and (dT)$_{70}$, (e) (dT)$_{70}$ and SSB-A, and (f) (dT)$_{70}$ and wtSSB. (a) SSB-A (10 μM), a homo-tetrameric SSB variant containing an additional six amino acids, TGASGT, at the C-terminus of each subunit, was titrated into RecO (1 μM) in buffer BTP (pH 7.0, 200 mM NaCl) at 15°C and (b) 25°C. SSB-A does not interact with RecO either in the presence or in the absence of ssDNA. (c) SSB-A (5.9 μM) was titrated into χ (1 μM) in buffer T (10 mM Tris-HCl, pH 8.1, 200 mM NaCl, 0.1 mM EDTA) at 25°C. SSB-A does not show binding to χ. (d) dT$_{70}$ (60 μM) was titrated into P65 (2 μM) in buffer BTP (pH 7.0, 20 mM NaCl) at 25°C. P65, the full-length C-terminal intrinsically disordered tail of SSB including the tip, does not interact with (dT)$_{70}$. (e) (dT)$_{70}$ (15 μM) was titrated into SSB-A (1 μM) in buffer T at 25°C. SSB-A

binds stoichiometrically to $(dT)_{70}$. Previous work with wtSSB binding to $(dT)_{70}$ under identical solution conditions observed a similar ΔH (-131 ± 2 kcal/mol), indicating that $(dT)_{70}$ binds identically to SSB-A and to wtSSB(1). (f) $(dT)_{70}$ (15 μM) is titrated into wtSSB (1 μM) in buffer BTP (pH 7.0, 200 mM NaCl). wtSSB binds stoichiometrically to $(dT)_{70}$. The difference in ΔH between the binding of SSB-A and wtSSB in panels (e) and (f) is due to the differences in the buffer and pH used in the two experiments(1).

Table S1: ITC Binding Data for Interaction of *E. coli* SSB C-Terminal Peptides and SIPs

		**Stoichiometry (N)**	**K (M^{-1})**	**ΔH (kcal/mol)**
**RecO**	P15	1.10 ± 0.01	$(1.8 \pm 0.2) \times 10^7$	-4.8 ± 0.1
	P31	0.96 ± 0.01	$(3.7 \pm 0.3) \times 10^7$	-5.4 ± 0.1
	P65	1.04 ± 0.01	$(1.7 \pm 0.1) \times 10^7$	-4.9 ± 0.1
**PriA**	P15	0.96 ± 0.30	$(2.5 \pm 0.5) \times 10^5$	-10.0 ± 0.4
	P31	1.15 ± 0.14	$(4.6 \pm 0.6) \times 10^5$	-8.2 ± 1.2
	P65	1.12 ± 0.07	$(5.0 \pm 0.4) \times 10^5$	-8.5 ± 0.7
**DNA Pol III (χ)**	P15	1.06 ± 0.07	$(3.1 \pm 0.2) \times 10^5$	-8.7 ± 0.8
	P31	0.90 ± 0.06	$(2.6 \pm 0.1) \times 10^5$	-10.3 ± 0.8
	P65	0.93 ± 0.06	$(6.5 \pm 0.7) \times 10^5$	-10.2 ± 0.9
**PriC (10 mM NaCl)**	P15	0.96 ± 0.05	$(1.5 \pm 0.2) \times 10^6$	-2.8 ± 0.2
	P31	1.01 ± 0.07	$(1.3 \pm 0.2) \times 10^6$	-2.3 ± 0.2
	P65	1.10 ± 0.11	$(1.0 \pm 0.2) \times 10^6$	-2.3 ± 0.5

K = observed association equilibrium constant, ΔH = enthalpy change

Table S2: ITC Binding Data for Replicate Measurements of Interaction of E. coli SSB or SSB C-Terminal Peptides and SIPs

	**Stoichiometry (N)**	**K (M^{-1})**	**ΔH (kcal/mol)**	**ΔH std. dev.**	**ΔG° (kcal/mol)**	**ΔG° std. dev.**	**TΔS° (kcal/mol)**	**TΔS° std. dev.**
**P15-RecO**	1.10 ± 0.01	$(1.8 \pm 0.2) \times 10^7$	-4.8 ± 0.1	0.14	-9.9 ± 0.1	0.07	5.1 ± 0.1	0.07
	1.05 ± 0.01	$(2.2 \pm 0.2) \times 10^7$	-5.0 ± 0.1		-10.0 ± 0.1		5.0 ± 0.1	
**P31-RecO**	0.96 ± 0.01	$(3.7 \pm 0.3) \times 10^7$	-5.4 ± 0.1	0.21	-10.3 ± 0.1	0.02	4.9 ± 0.1	0.23
	0.98 ± 0.01	$(3.9 \pm 0.2) \times 10^7$	-5.1 ± 0.1		-10.3 ± 0.1		5.2 ± 0.1	
**P65-RecO**	1.04 ± 0.01	$(1.7 \pm 0.1) \times 10^7$	-4.9 ± 0.1	0.44	-9.8 ± 0.1	0.36	4.9 ± 0.1	0.75
	0.84 ± 0.01	$(5.5 \pm 1.1) \times 10^7$	-4.1 ± 0.1		-10.5 ± 0.1		6.4 ± 0.2	
	0.96 ± 0.01	$(3.9 \pm 0.6) \times 10^7$	-4.8 ± 0.1		-10.3 ± 0.1		5.5 ± 0.1	
**P65-χ**	0.93 ± 0.06	$(6.5 \pm 0.7) \times 10^5$	-10.2 ± 0.9	0.28	-7.9 ± 0.1	0.28	-2.3 ± 0.9	0.01
	1.02 ± 0.10	$(3.4 \pm 0.2) \times 10^5$	-9.8 ± 0.6		-7.5 ± 0.1		-2.3 ± 0.6	
**P65-PriA**	1.12 ± 0.07	$(5.0 \pm 0.4) \times 10^5$	-8.5 ± 0.7	0.14	-7.8 ± 0.1	0.21	-0.7 ± 0.7	0.35
	1.01 ± 0.10	$(3.2 \pm 0.2) \times 10^5$	-8.7 ± 1.0		-7.5 ± 0.1		-1.2 ± 1.0	
**SSBΔ130-RecO**	3.80 ± 0.10	$(2.0 \pm 0.3) \times 10^6$	-1.8 ± 0.6	0.35	-8.9 ± 0.1	0.12	7.1 ± 0.6	0.48
	4.04 ± 0.10	$(1.5 \pm 0.1) \times 10^6$	-2.3 ± 0.1		-8.7 ± 0.1		6.4 ± 0.1	

K = observed association equilibrium constant, ΔH = enthalpy change, ΔH* = ΔH/4; ΔH per tip

Table S3: ITC Binding Data for Interaction of *E. coli* SSB C-Terminal P15 and RecO in buffer BTP

pH	[NaCl] (mM)		Stoichiometry (N)	K (M^{-1})	ΔH (kcal/mol)	ΔG° (kcal/mol)	TΔS° (kcal/mol)
8.0	50	25ºC	1.10 ± 0.01	$(1.8 \pm 0.2) \times 10^7$	-4.8 ± 0.1	-9.9 ± 0.1	5.1 ± 0.1
8.0	200		0.97 ± 0.02	$(2.9 \pm 0.3) \times 10^6$	-4.0 ± 0.1	-8.8 ± 0.1	4.8 ± 0.1
7.0	200	15ºC	1.04 ± 0.13	$(1.7 \pm 0.8) \times 10^6$	-2.4 ± 0.2	-8.2 ± 0.3	5.8 ± 0.3
		25ºC	1.07 ± 0.10	$(9.0 \pm 2.3) \times 10^5$	-6.1 ± 0.2	-8.2 ± 0.1	2.2 ± 0.2
		37ºC	0.97 ± 0.10	$(4.9 \pm 0.1) \times 10^5$	-8.2 ± 0.1	-8.0 ± 0.2	0.22 ± 0.8

K = observed association equilibrium constant, ΔH = enthalpy change

References for Supplementary Material

1. Kozlov, A.G. and Lohman, T.M. (2006) Effects of monovalent anions on a temperature-dependent heat capacity change for Escherichia coli SSB tetramer binding to single-stranded DNA. *Biochemistry*, **45**, 5190-5205.
